# Human Resilience and Pain Coping Strategies: A Review of the Literature Giving Insights from Elite Ultra-Endurance Athletes for Sports Science, Medicine and Society

**DOI:** 10.1007/s40279-025-02277-4

**Published:** 2025-07-17

**Authors:** Carole A. Paley, Mark I. Johnson

**Affiliations:** 1 Academic Unit of Palliative Care, Leeds Institute of Health Sciences, University of Leeds, Worsley Building, Clarendon Way, Leeds, West Yorkshire LS2 9NL UK; 2 Centre for Pain Research, School of Health, Leeds Beckett University, Leeds, West Yorkshire LS1 3HE UK

## Abstract

**Background:**

Elite ultra-endurance athletes face extreme physical and psychological challenges, often enduring prolonged pain, fatigue and adverse environmental conditions. This article explores the pain coping strategies these athletes employ and highlights parallels with chronic pain populations. Through a combination of genetic predispositions, physiological conditioning and psychological resilience, ultra-endurance athletes tolerate prolonged and severe bodily pain and discomfort. Resilience and self-efficacy are crucial traits, allowing athletes to persist in extreme conditions. Adaptive coping strategies such as mindfulness, enhanced interoception and emotional regulation help athletes navigate pain whilst optimising performance.

**Methods and key findings:**

We searched five electronic databases for the literature on ultra-endurance activities and key psychological concepts, extracting significant themes. We draw on parallels between ultra-endurance athletes and individuals with chronic pain. Both groups benefit from similar coping mechanisms, including acceptance of pain, reinterpreting discomfort as a positive experience and using cognitive strategies. Ultra-endurance athletes, however, often reframe pain as part of the reward system associated with achievement, which differs from the distress-driven narrative of chronic pain populations. This allows athletes to achieve personal and competitive goals in the face of severe physical discomfort. We also explore the perceived locus of control and how this can drive a positive and reward-driven appraisal of bodily discomfort.

**Conclusions:**

Understanding the psychological processes and mental strategies that enable athletes to perform in such extreme conditions offers valuable insights into pain management and performance, emphasising the role of mental resilience, mindfulness and cognitive reappraisal. This demonstrates the potential for improving pain tolerance and mental well-being through adaptive mental strategies and focused training.

## Key Points

**Table:** 

Ultra-endurance events require athletes to endure extreme physical and psychological challenges. Mental toughness, or self-efficacy, plays a critical role in overcoming these challenges, and athletes may develop unique pain coping mechanisms to manage discomfort and continue performance.
Endurance athletes may have more efficient neurophysiological mechanisms for pain inhibition, including descending inhibition of nociceptive neural input and reduced brain activity in areas associated with pain processing. It is likely that endocannabinoids also play a role in modulating pain.
Mental toughness and self-efficacy are essential for elite performance in ultra-endurance events. Adaptive strategies such as mindfulness, visualisation and emotional regulation help in maintaining focus, motivation and performance under extreme conditions.
Interoception plays a crucial role in performance. Ultra-endurance athletes learn to manage or dissociate from physical signals such as pain, fatigue and hunger, allowing them to push past normal limits. Training interoception helps athletes optimise performance while minimising injury risks.
It is suggested that pain can be part of a ritualistic transition process, or a ‘rite of passage’, as part of socialisation process with peer groups. Pain narratives provide a unifying experience within the group.

## Background

Ultra-endurance events are typically defined as activities of over 6 h duration [1], or in the case of running, any distance longer than the traditional 42.2-km (26.2-mile) marathon. They encompass single- or multi-day events, incorporating punishing conditions such as mountainous terrain, extremes of heat or cold, and long periods of complete solitude. Examples include the 100-mile Ultra-Trail du Mont-Blanc, the Marathon des Sables 6-day, 154-mile stage race through the Sahara Desert, the Tour Divide cycle race of 2700 miles down the US Rocky Mountains, The Norseman Xtreme triathlon and the Swedish Vidösternsimmet 21-km open-water swim.

Ultra-endurance athletes must be able to withstand extreme physical and psychological demands. Intensive training and competition require athletes to endure severe body discomfort, often in adverse weather conditions, including musculoskeletal pain, fatigue, sleep deprivation, hunger, thirst and injury, together with the unrelenting psychological demands of completing and competing in the event [2, 3]. Whilst individual genotypes [4] and neurophysiological/biomechanical characteristics [5] account, at least in part, for superior performance at such extremes, it has also been suggested that superior ‘mental toughness’, or self-efficacy, enables athletes to overcome extremes of physical adversity [2, 6, 7]. Personality profiles have also been shown to affect the perception, and tolerance, of pain [8], although it is unclear whether this is a result of endurance training, owing to a process of natural selection, or both. Likewise, the ability to overcome boredom during long races is an important attribute requiring significant mental effort [9]. Through evolutionary processes humans have become physiologically adapted for endurance [10], although the actual limits to this capacity, especially where individuals choose to participate, are probably set by an attitude of mind [11].

The aim of this review is to investigate the mechanisms of human resilience and pain management as demonstrated by elite ultra-endurance athletes. By synthesising the existing literature, this article seeks to identify key strategies and practices that contribute to their exceptional endurance and mental fortitude. The insights gained will be applied to enhance understanding in sports science, medicine and societal contexts, with the goal of improving performance, well-being, and adaptive coping strategies in both athletic and non-athletic populations.

## Methods

We conducted a search using several electronic databases; MEDLINE (via OVID), CINAHL, PsychINFO, SportDiscus and The Cochrane Database of Systematic Reviews using keyword searches, adapted for each database in the following string: ultra-marathon OR ultramarathon OR ultra-runner OR ultrarunner OR ultra-distance OR ultradistance OR ultra-endurance OR ultraendurance OR ultra-marathoner OR ultramarathoner OR ultra-marathons OR ultramarathons OR ultra-runners OR ultrarunners. We also searched the electronic databases for literature using broad psychological traits including ‘mind states’, mental toughness’, ‘self-efficacy’ and ‘interoception’ and included the key literature relating to chronic pain mechanisms.

All types of literature including randomised controlled trials, non-controlled trials, systematic or narrative reviews, and case studies were included. We also included studies using quantitative, qualitative or mixed methodologies.

We searched and selected articles that were relevant or of particular interest to our aim and organised the literature critically analysing the methodology, findings and relevance of each article to identify common themes and gaps, and highlighting key strategies and practices that contribute to the exceptional endurance and mental fortitude. This approach allowed us to draw out insights and implications for pain coping in the context of chronic pain. Many articles did not differentiate between pain intensity and unpleasantness so we decided not to distinguish between the two aspects. Pain is inherently subjective, and separating these dimensions may oversimplify the complex nature of pain experiences, particularly under extreme physical exertion or exhaustion. Higher pain intensity typically leads to greater unpleasantness, underscoring their interconnectedness and the dynamic interplay between them.

## Key findings and discussion

### Characteristics of Athletic Performance

Over many decades, researchers have tried to determine whether inherited genetic characteristics can reliably predict elite performance across different sporting disciplines [12]. In the case of endurance events such as marathon and ultra-marathon running, physiological characteristics such as cardiovascular capacity, muscle morphology and biomechanical factors are important, and all are influenced by genetically determined factors. It has been estimated that genetic influences could account for up to 66% of variance in athletic performance status [12]. However, it is unclear how important inherited characteristics are in terms of being accurately able to predict performance status, as many other influencing variables exist [13, 14]. It is also possible that the modification of genetically determined characteristics is possible through training and nutrition.

Some evidence suggests that endurance athletes possess superior pain modulation capabilities, indicating more efficient physiological inhibition of nociceptive neural activity associated with the emergence of pain [15–17]. Whether these capabilities are inherited, learned or developed through intensive training is unclear, although it has been suggested that even in non-athletes, an ability to develop a mindful acceptance of pain or an ability to re-focus and selectively direct attention (e.g., focusing on each breath) can be effective [18]. It is likely that there is a range of variables influencing the ability to withstand prolonged exercise-related pain, including the overriding influence of modifiable environmental factors.

The physiological, psychological and environmental components characterising endurance performance can be separated into their various elements to help disentangle the many influences on performance capacity. In Table 1, we identify which elements of these are essentially fixed, such as genetic characteristics, and those which could potentially be learned, modified and developed through training and other influences. Table 1 summarises how each performance component may be broken down into broad elements; physiological and psychological. Identifying each element and its potential for modification helps contextualise our later discussion on pain modulation and coping strategies in elite ultra-endurance athletes.

**Table 1:** Characteristics of athletic performance

Component	Origin	Elements	Modification potential
Evolution	Origin of human species, adapted for survival	Energy allocation, e.g. fight-or-flight response	*Enhancement/inhibition of some evolutionary characteristics might be possible with training* Reduced fear of pain or acceptance of it inhibits fight-or-flight response
Genotype	Genetic profile (inherited characteristics)	Biological sex Anatomical structure Somatotype Pain sensitivity	*Essentially fixed, with potential to enhance and maximise existing genetic characteristics and modify gene expression* Psychological methods to enhance pain-coping mechanisms
Phenotype	Expression of genotype, which may be influenced by environmental, psychological and sociological factors Geographic ancestry might also be important	Endurance capacity Muscle morphology Biomechanics Connective tissue characteristics Haemodynamic characteristics Metabolism Body composition	*Modifiable within the limits of the genotype* Body composition Joint flexibility Artificial adjustment of biomechanics e.g., running shoes, foot orthoses Aerodynamic equipment Specialist clothing Nutrition/diet
Psychology	Genetic profile Phenotypical influences	Personality traits Self-efficacy/mental toughness Interoception Motor inhibitory capacity Perception/tolerance of pain Cognitive function	*Modifiable with training* Training effects on ability to tolerate pain/discomfort Increased interoceptive accuracy through training improves regulatory ability Override dominant motor responses Improved cognitive function Mental training
Environment	Socioeconomic background Culture/religion Geographical location Education Exposure to sport/activity (parents, peers)	Availability of finance, transport and equipment Cultural/religious norms Topography, climate, transport network Access to activity in communities, schools, colleges and universities Parental/family participation and/or encouragement Peer or social group Facilities for training Availability of coaching	*Highly modifiable within available resources* Improved public/community services and infrastructure Community sports facilities and clubs Family participation, e.g. taster sessions Improved school sports facilities and grounds, after school clubs Community clubs/leagues Facilities and coaching for under-represented groups

### Pain Modulation in Ultra-Endurance Athletes

#### Neuromodulation and Pain Processing

The ability to cope with pain is essential in athletes facing prolonged and often severe bodily discomfort and pain during training and competitions, often for many hours and sometimes over periods of days or even weeks. Pain is thought to be inhibited by the activation of endogenous mechanisms in the central nervous system, for example, descending inhibition of nociceptive neural input, and it has been suggested that endurance athletes possess an enhanced ability to activate these endogenous mechanisms [19]. A recent functional magnetic resonance imaging study using a noxious heat stimulus to elicit ‘experimental’ pain demonstrated that descending inhibition of nociceptive neural input was more efficient in endurance athletes as compared with non-athletes [16]. The study also revealed reduced activation of brain regions associated with the processing of nociceptive input in the endurance athlete group, suggesting differences in neurophysiological mechanisms associated with the emergence of pain as a percept from complex interactions within the nervous system, arising from the brain’s processing of nociceptive information. It is possible that ultra-endurance athletes can pay less attention to the pain which is related to sporting endeavours (e.g. pain associated with sporting effort [physical excursion] and/or actual or potential sporting-related injury), thus enabling them to cope better with intense and prolonged bodily discomfort. Furthermore, neuromodulation processes that act as warning signs indicating actual or potential tissue damage could also be overridden, thus giving insight into how athletes are able to continue activities with severe injuries [11, 20]. Endocannabinoids are also thought to play a role in the reduction of pain perception and the regulation of inflammation and have recently been linked to an exercise-induced euphoria, which was previously thought to be mediated primarily by endorphins [21, 22].

Ultra-athletes have been found to have reduced pain-avoidance behaviours and pain-related anxiety, which at least partially mediates both tolerance to pain and termination of pain-related activities [8, 23]. It is possible that these abilities are generated through prolonged and intensive endurance training [24]. Fear or anxiety about pain is associated with reduced activity in endogenous inhibition of nociceptive input in the central nervous system, interfering with the ability to manage pain effectively, and this can occur in both novice athletes and non-athletic populations [25]. A recent review also suggested that recreational runners with high anxiety experienced a longer recovery process from lower limb injuries [26]. There might also be anxiety about causing permanent tissue damage in people not taking part in intense or prolonged physical activity, for example, fear avoidance of pain-related movement.

The findings of studies using experimental pain and brain imaging techniques suggest that physical training alongside specific mental training strategies lowers the fear of pain and generates an expectation and acceptance that it is ‘safe’ to experience pain, resulting in a higher tolerance to experimentally induced pain [15, 16]. Expectation and acceptance of bodily discomfort promotes an ability to cope with the emotional unpleasantness experienced during a long and punishing event. Research has indicated that athletes who have realistic expectations of how they will feel during an event report pleasant emotional responses to exercise [27]. Another study demonstrated that greater trait emotional intelligence was associated with pleasant emotions during a 6-day multi-stage running event [28].

In a study using an experimentally induced, noxious stimulus paradigm, triathletes were found to have higher pain tolerance and better coping with the fear of pain than non-athlete controls [15]. The triathletes also exhibited enhanced conditioned pain modulation, also known as diffuse noxious inhibitory control, and a proxy of the ‘efficiency’ of the descending inhibitory modulation system to inhibit nociceptive input when an additional noxious stimulus is simultaneously applied at a different body site, i.e., “pain inhibiting pain” [29]. Reduced activity of the descending inhibitory modulation system manifesting as a lower conditioned pain modulation response has been associated with chronic primary pain conditions such as fibromyalgia [30]. The implications of an increased conditioned pain modulation and less likelihood to avoid severe pain could leave endurance athletes more susceptible to tissue damage [17, 19, 31].

#### Interoception

In 2003, Craig introduced his theory of homeostatic emotion, which suggested that pain is an emotional drive that alerts the body to potential harms and motivates behaviours that will restore balance [32, 33]. The theory emphasised the importance of pain as both a sensory and emotional (affective) experience that motivates humans to protect the body and promotes healing behaviours, thus retaining homeostasis. This sense of the physiological condition of the body (i.e., “how do I feel?”) is described as interoception, or our sense of the internal physical and emotional state of the body, either subconsciously or consciously interpreted [34]. Disruption of interoception results in a dysregulation of emotional responses to pain [35]. This mechanism is therefore important in both athletes and non-athletes.

Interoceptive disruption occurs in chronic pain whereby individuals ‘feel’ something is wrong with their body, even when there may be an absence of observable tissue damage. This may trigger behavioural changes such as fear avoidance of painful movement or excessive guarding of painful body parts, in an attempt to prevent perceived harm [32, 33]. These behavioural changes in themselves can exacerbate fear avoidance of pain behaviour creating a feedback loop, contributing to the anxiety associated with chronic pain.

Interoception can be shaped by exercise training and this improves the regulation of exertion and accurate monitoring of fatigue in a feedback loop involving constant processing and appraisal of interoceptive inputs [34]. Enhanced interoceptive accuracy results primarily from neuroplastic changes in the insula and surrounding networks [36]. When the ability to appraise interoceptive signals is effective, it can help regulate emotional reactivity to different situations and thus contributes to well-being [37]. Chronic physical training, as in ultra-endurance athletes, results in more accurate interoceptive appraisal, although elite athletes train themselves either to manage or dissociate from interoceptive signs such as pain, fatigue and hunger, allowing them to push beyond normal physical limits [34]. Studies have shown that ‘mind-over-body’ beliefs, in combination with perceived limits of endurance, are important factors in performance [38, 39].

These adaptations, which occur as a result of training, involve a combination of mental strategies, physiological conditioning and a focus on long-term goals, which help athletes navigate the intense demands of ultra-distance events. However, this does not mean athletes completely ignore these signals; many successful ultra-athletes learn to interpret and respond to them in ways that help optimise performance while minimising injury risk. Mehling et al. proposed that interoceptive accuracy and interoceptive awareness were different and were dependent upon attention [40]. The integration of interoceptive information with physical and emotional input [41] provides some explanation of how ultra-athletes can push through seemingly impossible limits and offers a possible mechanism of action for some of the popular mind–body therapies and mental training interventions [40, 42].

#### Mind States and Mental Toughness

The ability to tolerate and cope with pain is a key feature of elite endurance performance [43]. Mental toughness is described as psychological resilience, or the ability to cope with various stressors, including pain and adversity. It is also associated with self-efficacy, persistence and being able to manage failure [2, 44, 45]. Several studies of ultra-marathon athletes have revealed the existence of enhanced mental toughness and self-efficacy [2, 6, 46, 47]. A study on athletes who had completed the 250-km Marathon des Sables over 6 days across the Sahara Desert found distinct character profiles in that athletes had higher levels of extroversion and openness to experience as compared with population norms [6]. There is a suggestion that the supra-threshold levels of mental toughness and associated self-efficacy required to achieve success in ultra-marathon events will respond to psychological skills training, but this is, as yet, unclear [2]. However, a recent study suggested that for elite ultra-endurance athletes, a criterion level of mental toughness and self-efficacy is a pre-requisite in order to be able to cope with the level of training and performance needed [48]. Beyond this level, other factors including mental state and psychological adaptations become more important [2, 49]. This would suggest that individuals already exhibiting higher levels of these attributes are more likely to self-select ultra-endurance sports.

#### Locus of Control

Locus of control plays a crucial role in how individuals respond to pain and other forms of bodily discomfort [50]. Successful ultra-endurance athletes are more likely to possess an internal locus of control, which is associated with active coping, resilience and higher levels of self-efficacy [2]. These individuals have better levels of acceptance of the pain and are less emotionally distressed by the pain, having greater psychological flexibility. In contrast, individuals with an external locus of control are more likely to develop fear avoidance behaviours and hypervigilance [51]. They are less likely to engage with behaviours that are perceived to be painful, including self-care activities and are more likely to rely on passive forms of control, such as medication [52]. Conversely, in athletes, an internal locus of control is associated with increased mental toughness and higher levels of perseverance and control under pressure, which are crucial traits for excelling in ultra-endurance sports [53]. These endurance athletes are more proactive and able to effectively self-regulate [54], which enables them to manage pain and fatigue more effectively, and handle adversity with resilience.

### Strategies for Coping with Pain in Ultra-Endurance Events

#### Adaptive Coping Strategies

A study exploring pain during multi-stage ultra-marathons found that athletes who predominantly used adaptive coping strategies (e.g., mindfulness, visualisation, pacing) were better able to manage pain interference than those who used maladaptive strategies (e.g., pain catastrophising, rumination, avoidance) [55]. The ability to cope with pain, as observed in chronic pain populations, is associated with positive effects on pain interference and this might be possible to enhance through mental training [56]. As already discussed, pain that is anticipated is less likely to cause distress than situations where pain is unexpected or is associated with some uncertainty, as found in chronic pain conditions [55, 57]. It has also been suggested that where pain experiences have a positive context, (e.g., exhilaration, sense of achievement, reward or overcoming adversity), the anticipation of intrinsic reward promotes positive emotional responses to it. Acceptance of pain and even taking enjoyment or satisfaction from it has been shown to help athletes to withstand bodily discomfort. According to Bourke [58], it is possible to change the meanings ascribed to pain, and in this way, previously negative sensations can become positive. Parallels to this have been seen in recent pain management initiatives with non-athletic populations, where education, understanding, and acceptance of pain are keystones of the approach and individuals with persistent pain are given the chance to express their pain and assign new meanings to it [59–61]

Cognitive function has also been shown to predict performance in ultra-trail races because athletes have an ability to filter out irrelevant and distracting information [62]. A recent literature review exploring the psychological indicators of success in ultra-running suggested that the presence of high levels of self-efficacy and cognitive strategies to maintain mood stability could be vital to success [63]. The same review also found that psychological skills such as self-talk, imagery, and goal setting were important and traits such as emotional intelligence enable athletes to self-regulate cognitive processes during situations that are challenging. Other mental strategies and techniques employed by ultra-athletes include emotional regulation and positive reappraisal strategies [3, 64].

#### Mindful States

Evidence from non-athletic populations suggests that mindful states can result in the modulation of persistent pain [65, 66]. In a study of recreational cyclists, it was found that mental toughness and mindfulness were positively related with each other, and that mindfulness was negatively associated with pain catastrophising [67]. Mindfulness is a state of adaptive coping and can enhance mental toughness by promoting emotional regulation.

In a qualitative phenomenological study of ultra-marathon runners in 2014, participants reflected that they used a process to divert their attention away from the pain and more towards execution of movements, including concentrating on the rhythm of running [68]. Other adaptive cognitive strategies included self-talk, imagery and attentional focus control, but dissociating from the pain, i.e. by reducing conscious attention toward incoming nociceptive stimuli and instead concentrating on external, or environmental cues, was not effective. This finding contrasted with earlier studies [69] that found that athletes successfully used dissociative strategies. However, dissociation techniques could distract athletes from interoceptive appraisal and provide emotional detachment at a time when these inputs are necessary for self-regulation and monitoring.

A further study explored the effects of a mindfulness programme, including using a ‘body-scan’ technique, on oxygen consumption and running economy and the flow experience during different loci of focus, finding that mindfulness training improved running economy and enhanced flow experience [70].

#### Spirituality, Resilience and Social Bonding

Using a temporal framework for describing the experiences of ultra-endurance athletes, a qualitative study described intense bodily suffering as ‘cathartic’ and likened it to a ‘beautiful suffering’, describing a new level of consciousness and joy through pain and hardship [71]. Other authors have described spiritual fulfilment, enlightenment [72] and even the mysticism of running [73]. These descriptions are resonant of the Medieval ascetics and other religious figures who were able to withstand extreme self-inflicted pain and hardship in order to achieve enlightenment [71, 74]. In 2018, Díaz-Gilbert referred to ultra-runners as “ascetic athletes” who defy normal limits of human tolerance, likening them to the early Christian ascetics who experienced “… exhilarating suffering and joy…” as a result of their self-inflicted torture [75]. Although this is a view from a devout Christian perspective, it reflects the findings of other authors who claim that the mind-set of the ultra-athlete incorporates an expectation, an acceptance of, and even pleasure, in extreme bodily discomfort and hardship [76, 77]. Atkinson describes fell running (i.e., running on the hills or mountains) as “gritty play”, exploring how runners relish their self-inflicted hardship in a “sweaty camaraderie” [76]. It has also been suggested that the pleasure derived from pain in runners is part of a ritualistic transition process, or ‘rite of passage’, which is achieved through a learning process and as part of socialisation with running peer groups [77]. In this way, pain narratives provide a unifying experience within the group [58]. Pain, as negatively experienced by novice runners, is thus reinterpreted as a positive experience, which is both pleasurable and desirable [77]. Johnson et al. discuss the insidious nature of the societal pain narrative, which is grounded in a destructive damage-loaded war-mongering metaphor, which in many ways contrasts with the constructive positive experience that bonds together the ultra-marathon ‘tribe’ [78].

The embodied experiences of ultra-athletes and the intrinsic rewards of overcoming severe pain and hardship provide some explanation as to why these athletes put themselves through the rigours of training and competition. The euphoria described by some athletes, often referred to colloquially as the ‘runner’s high’ is likely to occur after a physically testing event or training session. Physiologically, increased levels of endogenous opioids and endocannabinoids circulating within the central nervous system are associated with this euphoria, mediation of stress and enhanced pain tolerance [15, 22, 79]. However, it should be noted that the same mechanisms might also contribute to excessive exercise, or an exercise addiction, which can be injurious to both mental and physical health [80, 81].

## Conclusions

In this article, we have explored the physical and psychological challenges faced by ultra-endurance athletes, focusing on their ability to endure pain, fatigue, and adversity during long intense events and overcome extreme environmental challenges. The ability to handle pain is essential for success and research suggests that ultra-athletes possess enhanced pain modulation capabilities. Ultra-endurance sports require athletes to cope with extreme physical discomfort, mental stress and isolation. These athletes exhibit superior pain tolerance and mental toughness, which may be linked to their physical conditioning, genetic factors, and psychological traits such as self-efficacy and resilience. Mental toughness and self-efficacy are key psychological traits, and an enhanced interoceptive ability plays a crucial role in regulating responses to pain and fatigue. The literature suggests that resilience to stress, pain and adverse conditions can be developed through persistent physical and mental training. Techniques such as mindfulness, emotional regulation and positive reappraisal help reframe pain as a positive and ultimately rewarding experience. Athletes also demonstrate a heightened interoceptive awareness, which aids regulation of their physical and emotional responses to pain and hardship.

We have also explored the role of psychological factors such as the locus of control (internal vs external) in managing pain and stress. Athletes with an internal locus of control tend to cope more effectively with pain, demonstrating higher levels of perseverance and self-regulation. Where individuals feel that they have mastery over their pain and bodily discomfort, this leads to intrinsic satisfaction and reward, camaraderie and peer-group acceptance and becomes a positive experience. Adaptive coping strategies, including cognitive techniques such as visualisation and pacing, also improve performance and pain management.

Finally, the importance and influence of cultural and social factors were discussed, including the effect of peer groups, which transform the meaning of pain into a representation of resilience and success. It has been suggested that athletes experience a sense of spiritual fulfillment or “beautiful suffering” as they push through extreme physical and mental challenges, which have been described as a ‘rite of passage’.

Throughout this article, we have revealed parallels with non-athletic chronic pain populations. A key factor determining an individual’s perception of pain is the locus of control. We suggest that pain, whether from ultra-endurance sports or chronic medical conditions, can be managed through a blend of adaptive coping strategies, and mental and social reframing. This can be learned and developed within the environmental and social conditions that reframe pain within a positive context. Mastering pain through mental and social strategies turns it into a symbol of resilience and success, thus emphasising the transformative power of mental and social approaches in redefining pain.

## Declarations

### Availability of Data and Material

Materials are available on request from the corresponding author.
